# Evolutionary Algorithm Based Capacity Maximization of 5G/B5G Hybrid Pre-Coding Systems

**DOI:** 10.3390/s20185338

**Published:** 2020-09-17

**Authors:** Salman Khalid, Waqas Bin Abbas, Hyung Seok Kim, Muhammad Tabish Niaz

**Affiliations:** 1 Department of Electrical Engineering, National University of Computer and Emerging Science, Islamabad 44000, Pakistan; i171402@nu.edu.pk (S.K.); waqas.abbas@nu.edu.pk (W.B.A.); 2 Department of Intelligent Mechatronics Engineering, Sejong University, Seoul 05006, Korea

**Keywords:** 5G/B5G communication systems, achievable rate, evolutionary algorithms, hybrid pre-coding, interference cancellation, millimeter wave, wireless communication systems

## Abstract

Hybrid pre-coding strategies are considered as a potential solution for combating path loss experienced by Massive MIMO systems operating at millimeter wave frequencies. The partially connected structure is preferred over the fully connected structure due to smaller computational complexity. In order to improve the spectral efficiency of a partially connected hybrid pre-coding architecture, which is one of the requirements of future 5G/B5G systems, this work proposes the application of evolutionary algorithms for joint computation of RF and the digital pre-coder. The evolutionary algorithm based scheme jointly evaluates the RF and digital pre-coder for a partially connected hybrid structure by taking into account the current RF chain for computations and therefore it is not based on interference cancellation from all other RF chains as in the case of successive interference cancellation (SIC). The evolutionary algorithm, i.e., Artificial Bee Colony (BEE) based pre-coding scheme outperforms other popular evolutionary algorithms as well as the SIC based pre-coding scheme in terms of spectral efficiency. In addition, the proposed algorithm is not overly sensitive to variations in channel conditions.

## 1. Introduction

For capacity enhancement of upcoming 5G systems various physical layer [1,2,3,4,5] and network densification techniques [6,7,8,9] are investigated. The emergence of millimeter-wave (mmW) with massive multiple-input multiple-output (MIMO) is considered as a promising technique for the upcoming 5G/beyond 5G (B5G) wireless communication systems [10,11,12], as they can provide an increase in both the available bandwidth and the spectrum efficiency [13,14]. However, as per Friis Law, communication at such high frequencies results in high path loss, and therefore omni directional transmission is not feasible due to considerable coverage reduction. However, vice versa the short wavelength associated with mmW frequencies enables to constitute a massive MIMO architecture using a large antenna array [15,16]. A massive MIMO architecture can provide sufficient gains by using pre-coding and beam-forming techniques to encounter signal attenuation caused by mmW frequencies [17,18]. For pre-coding, three main architectures are considered for millimeter wave communications, i.e., (1) a fully digital pre-coder, (2) an analog architecture, and (3) a hybrid pre-coder.

A fully digital pre-coder achieves the optimal performance for massive MIMO systems, however, it requires a separate radio frequency (RF) chain connected to each antenna element. As a consequence, for a millimeter wave massive MIMO system, where the base station (BS) will be equipped with a large number of antennas, the hardware complexity and the energy/power consumption of a digital pre-coding scheme becomes a serious concern [10,19]. One solution is an analogue beam-forming scheme, which is power efficient but does not provide the advantages of digital processing. To address the problem, the hybrid pre-coding technique constituting the digital pre-coding and the RF pre-coding has been proposed to optimize the number of RF chains. As both the energy/power consumption and the hardware complexity are reduced without much performance loss, the hybrid architecture is considered as an essential requirement for practical mmW massive MIMO systems [20]. In existing literature, two types of hybrid pre-coding structures are discussed: (a) A spectral efficient structure or a fully connected architecture, where all antennas are connected through phase shifters to each RF chain and (b) an energy efficient structure or a partially connected architecture, where limited number of antennas are connected to a RF chain. However, the joint design of a digital and RF pre-coder is still challenging.

In existing literature, many schemes are proposed to design the hybrid pre-coder. The hybrid pre-coder based on spatially sparse pre-coding was proposed in [21,22,23], in which the achievable rate optimization problem is formulated as a sparse approximation problem and is solved using the orthogonal matching pursuit (OMP) algorithm to achieve the near-optimal performance. The authors in [24] proposed the codebook based hybrid pre-coder design, in which a predefined code book is created and iteratively used to find the optimal hybrid pre-coder matrix. Hybrid beamforming design for a multi-user massive MIMO downlink transmission using hybrid regularized channel diagonalization (HRCD) scheme was proposed in [25]. The technique consists of the RF phase-only analog precoder with the linear digital (baseband) precoder. Pre-coding in a partially connected structure with emphasis on energy efficiency maximization by adopting successive interference cancellation (SIC) based technique is discussed in [26,27,28]. Results showed that SIC based scheme is performing very close to sparse pre-coding scheme and the optimal unconstrained partially connected hybrid pre-coder. Hybrid precoder design for a multi sub array architecture is discussed in [29]. Recently, Manifold Optimization (MO) and an evolutionary algorithm (i.e., Particle Swarm Optimization (PSO)) based techniques are investigated in [30,31,32]. In [33], authors proposed a joint design of the analog precoder and decoder using a Riemannian optimisation method based on Stiefel manifold (ROSt). The analog precoder minimizes the interference from same angle of arrivals (AoAs). For the digital precoder a minimum mean square error (MMSE) based solution is opted. Authors in [34] have utilized the signal to leakage noise ratio (SLNR) to design the analog pre-coder and the conventional zero forcing technique is used for the digital pre-coder. An algorithm based on singular value decomposition to design the hybrid pre-coder is discussed in [35].

Recently, the transceiver design aiming at energy efficiency maximization is also investigated. Authors in [36] have proposed a two stage hybrid transceiver design to maximize the energy efficiency involving multiple IoT devices. Pre-coding for fully connected sub arrays is considered in [37]. Authors in [38] has achieved rate maximization by performing the pre-coding and beamforming for a single user massive MIMO system with a partially connected hybrid structure. Both the pre-coder and beamformer is designed using alternating minimization technique. However, our work is mainly focused on the transmit pre-coder design and does not require any feedback from the receiver. In [39], authors proposed a pre-coder design aiming for high user density. Authors in [40] have developed pre-coder and beamformer for multi user MIMO systems equipped with large antenna arrays. The efficient pre-coder design for partially connected hybrid structure is not well investigated. Keeping in view the high energy efficiency of partially connected hybrid structure, in this paper we have investigated the spectrally efficient pre-coder design for partially connected hybrid structure. Researchers are also investigating the data driven based approaches for hybrid pre-coder design [41,42]. However, the constraint of large data set for training the machine learning algorithm poses a challenge. The utilization of data driven approaches for partially connected hybrid structure is left as a topic of future research.

While the pre-coding with a fully-connected structure is spectrally efficient, the partially connected hybrid pre-coding approach is practically more feasible due to energy efficient design and low complexity. However, the efficient design of a hybrid pre-coder for a partially connected structure is challenging [43]. The evolutionary algorithm based technique, which do not require SVD or channel inversion, are not well investigated and only the PSO based technique has been implemented showing promising results. Therefore, inspired by the efficiency of the PSO based technique, in this paper we have investigated other evolutionary algorithms. The motivation behind adopting the evolutionary algorithms for pre-coder design is their simple architecture. The evolutionary algorithms are governed by simple algebraic equations and algorithms do not require SVD or channel inversion. Moreover, since each RF chain is treated as a separate entity, the problem of error propagation, which is a concern in SIC based scheme, is eliminated. Moreover, since the literature lacks the spectrally efficient pre-coder design for partially connected hybrid structure, in this paper, we have presented a spectrally efficient design for pre-coder. The main contribution of this work is as follows:

- We investigated the performance of evolutionary algorithms for capacity maximization and the results show that the proposed artificial bee colony (BEE) technique outclass other evolutionary algorithms in terms of achievable rate.
- We characterize the performance of pre-coding algorithms for different numbers of RF chains. It is shown that for a higher number of RF chains (where antennas per RF chain is reduced) BEE outperforms the SIC based technique for achievable rate, and the performance gap increases with the number of RF chains. Moreover, when the number of RF chains is less (where antennas per RF chains is high) the performance of the BEE algorithm is comparable to the SIC based approach for achievable rate. However, BEE outclasses all other evolutionary algorithms in every scenario.
- The performance of the proposed BEE algorithm is also investigated in the case of imperfect channel state information (CSI). Simulations results verify that the proposed algorithm is not overly sensitive to channel state information (CSI) accuracy.

The rest of the paper is organized as follows. Section 2 describes the system model for a partially connected mmW hybrid structure. Section 3 explains the pre-coder design using evolutionary algorithms. Section 4 presents the simulation results and the conclusions are discussed in Section 5.

Notations: Matrices are represented as upper case bold face letters and vectors are represented as lower case bold face letters; Hermitian, inverse and determinant are represented as ${\left(.\right)}^{H}$, ${\left(.\right)}^{-1}$, and $\left|.\right|$, respectively, and the Frobenius norm is represented as ${∥.∥}_{F}$.

## 2. System Model

**Figure 1:**
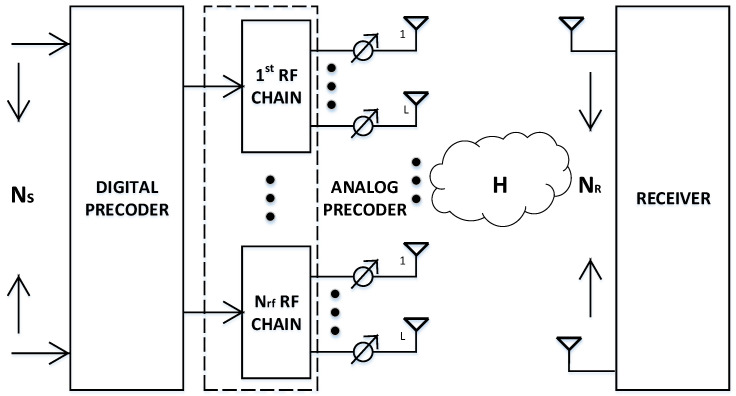
System Model.

We have considered the mmW massive MIMO system with the partially connected sub-array structure as shown in Figure 1, where the BS is equipped with $N_{T}$ transmit antennas, each having $N_{rf}$ RF chains and *L* antennas connected per RF chain to simultaneously transmit $N_{s}$ data streams (where $N_{s}=N_{rf}=N$) for the user, which is equipped with $N_{R}$ receive antennas. The $N_{s}$ data streams in the baseband are first precoded by an $N_{rf}$ × $N_{s}$ digital pre-coder D = diag [$d_{1}$, $d_{2}$, …, $d_{N}$], where $d_{n}$ ∈ *R* and is used for power allocation. Afterwards, data streams pass through $N_{rf}$ RF chains and each data stream is precoded again by an *L* × 1 RF pre-coder $a_{n}$ (*n = 1, …, N*) ∈$C^{L\times 1}$ realized by phase shifters having all elements with different phases but the same amplitude. After RF pre-coding, *L* antennas connected to the each RF chain transmits data from the respective data stream. The $N_{R}$ × 1 signal vector y received at the user is

(1) $$y=\sqrt{P_{av}}\mathrm{HADs}+n$$

where $P_{av}$ is the average received power, s is the transmitted signal vector, n = ${\left[n_{1},n_{2},\ldots ,n_{N}\right]}^{T}$ is independent and identically distributed (i.i.d) complex Gaussian noise, $CƝ(0,\sigma^{2})$, and H is a $N_{R}$ × $N_{T}$ channel matrix. Let F=AD be the hybrid pre-coding matrix of size NL×N, where A = [$a_{1}$, $a_{2}$, …, $a_{N}$], is a block diagonal RF pre-coding matrix consisting of *N* analogue weighting vectors.    

(2) $$A=\left(\begin{array}{cccc} a_{1} & 0 & \cdots & 0\\ 0 & a_{2} & \cdots & 0\\ \vdots & \vdots & \ddots & \vdots \\ 0 & 0 & \cdots & a_{N} \end{array}\right)$$

Similarly, D = diag [$d_{1}$, $d_{2}$, … , $d_{N}$] is a diagonal digital pre-coding matrix to perform the power allocation and expressed as

(3) $$D=\left(\begin{array}{cccc} d_{1} & 0 & \cdots & 0\\ 0 & d_{2} & \cdots & 0\\ \vdots & \vdots & \ddots & \vdots \\ 0 & 0 & \cdots & d_{N} \end{array}\right)$$

To model the mmW channel, we have followed the Saleh–Valenzuela channel model [44]. Due to the limited number of scatterers for mmW frequencies, the rich scattering channel model assumed for lower frequencies is not suitable for systems operating at mmW frequencies.

(4) $$H=\sqrt{\left(\frac{N_{T}N_{R}}{\epsilon K}\right)}\sum_{k=0}^{K}\eta_{l}a_{R}\left(\mu_{k}\right)a_{T}^{H}\left(\theta_{k}\right)$$

where *K* denotes the channel paths linked with limited scatterers, ϵ represents the pathloss, $\eta_{k}$ represents the complex gain associated with the *k*th path, symbols $a_{T}$ and $a_{R}$ represent the spatial signatures of the transmitter and the receiver, respectively. $\theta_{k}$ and $\mu_{k}$ represent the angle of departure (AoA) and the angle of arrival (AoD) of the *k*th path at the transmitter and at the receiver, respectively. The spatial signatures for a Uniform Linear Array (ULA) structure at the transmitter and the receiver can be expressed as,

(5) $$a_{R}\left(\mu \right)=\frac{1}{\sqrt{N_{R}}}{\left[1,e^{j\frac{2\pi }{\lambda }d\sin\left(\mu \right)},\ldots ,e^{j\left(N_{R}-1\right)\frac{2\pi }{\lambda }d\sin\left(\mu \right)}\right]}^{T}$$

(6) $$a_{T}\left(\theta \right)=\frac{1}{\sqrt{N_{T}}}{\left[1,e^{j\frac{2\pi }{\lambda }d\sin\left(\theta \right)},\ldots ,e^{j\left(N_{T}-1\right)\frac{2\pi }{\lambda }d\sin\left(\theta \right)}\right]}^{T}$$

where the signal wavelength is denoted by λ and the spacing between antenna elements is denoted by *d*.

## 3. Hybrid Pre-Coder Design Using Evolutionary Algorithm

The goal is to jointly design the RF pre-coder A and the digital pre-coder D that maximizes the achievable rate for the considered partially connected hybrid structure, which is expressed as,

(7) $$R=\log_{2}\left(I_{N_{R}}+\frac{\rho }{N_{rf}}HFF^{H}H^{H}\right)$$

here ρ is the signal to noise ratio (SNR). Now the hybrid pre-coding matrix F has to satisfy two constraints; C1: All the non-zero elements of the RF pre-coding matrix A should have the same amplitude to satisfy the unit norm constraint. C2: To meet the total power constraint, the Frobenius norm of F should be ${∥F∥}_{F}^{2}\leq N_{rf}$.

The above problem is a NL×N matrix optimization problem, which is tough to solve. Note that as F = $\left[f_{1},f_{2},\ldots ,f_{N}\right]$ is a block diagonal matrix, therefore the pre-coder design for different sub-arrays will be independent. This allows to break the above optimization problem into multiple sub optimization problems each of which only considers one sub-array.

In the following subsections, we will discuss the evolutionary algorithms, particularly, focusing on BEE. The reasons for choosing these algorithms are:

- Evolutionary algorithms require minimal tuning parameters, therefore these algorithms can be implemented for real-time applications.
- Evolutionary algorithms only requires the fitness function and do not require any differentiation, matrix inversion and Singular Value Decomposition (SVD), hence, resulting in reduced complexity [45].

### 3.1. Artificial Bee Colony Optimization

As per the argument given above, in each evolutionary algorithm, F is divided as $\left[f_{1},f_{2},\ldots ,f_{N}\right]$ and then the optimization problem for each $f_{i}$ is solved separately. Furthermore, each RF chain will be designed to maximize capacity. The achievable rate of the *n*th RF chain is optimized by designing the pre-coding vector $f_{n}$

(8) $$R_{n}=\log_{2}\left(1+\left(\rho \right)\times f_{n}^{H}H^{H}Hf_{n}\right)$$

We now highlight how the BEE algorithm selects the optimized pre-coding vector $f_{n}$. In BEE, the colony of artificial bees comprises three groups, employed, onlooker and scout bees (corresponds to sample space). Employed bees are associated (or employed) with specific food sources (corresponds to beam-forming vectors) and contains the information regarding the beam-forming vectors. Onlookers and scouts are termed as unemployed bees. Onlooker bees watch the activity of employed bees to select (or establish) a beam-forming vector and scout bees search for new beam-forming vector randomly. Initially, random beam-forming vectors $f_{n}$ of size L×1 equal to the number of employed bees are generated. Furthermore, their fitness (i.e., achievable rate) is evaluated using (8). The beam-forming vector that maximizes the objective function (achievable rate) is termed as the best solution ($f_{best}$). Thereafter, a search phase is initiated by employed bees and onlooker bees. Each employed bee produces a new beam-forming vector $f^{new}$ from the current one $f^{current}$. For the *i*th bee a new beam-forming vector can be computed as.

(9) $$f_{i}^{new}=f_{i}^{curr}+\phi ⊙\left(f_{i}^{curr}-f_{j}^{curr}\right)$$

where ⊙ represents the Kronecker product, $f_{j}^{curr}$ is a randomly selected beam-forming vector (i≠j), ϕ is a random vector (L×1) within [−1,+1] and $f_{i}^{new}$ is a new beam-forming vector of the *i*th employed bee. Once a new beam-forming vector is generated, it is compared with the previous one based on achievable rate. If the achievable rate of the new beam-forming vector is better than the old one, it will replace the old one, otherwise the old one is retained.

Employed bees share their experiences with onlooker bees and then onlooker bees probabilistically choose the beam-forming vectors depending upon their fitness (achievable rate). For this purpose, a fitness based selection (roulette wheel selection) can be used [44]. The probability value $p_{n}$ with which beam-forming vector is chosen by an onlooker bee can be calculated by using the expression

(10) $$p_{n}=\frac{fitness(f_{i})}{\sum_{n=1}^{N}fitness\left(f_{n}\right)}$$

where $fitness(f_{i})$ is the achievable rate corresponding to the *i*th beam-forming vector. Now obviously more the fitness, the higher is the probability that the *i*th beam-forming vector will be selected. This beam-forming vector is termed as $f_{i}^{curr}$ for *i*th onlooker bee. After that, the onlooker bee produces a new beam-forming vector as per (9) and if that results in a higher achievable rate than it replaces the current beam-forming vector.

In an iteration, after every employed and onlooker bee completes the respective searches, the BEE algorithm checks employed bees whose achievable rate cannot be improved through a predetermined number of trials. Such bees become scouts and their beam-forming vectors are abandoned. Then, the converted scouts generate new beam-forming vectors, randomly again.

Lastly, the achievable rate of the population is compared with the achievable rate of $f_{best}$. Moreover, the beam-forming vector that produces the best achievable rate is termed as $f_{best}$ and is the desired beam-forming vector for respective RF chain. The complete algorithm is summarized in Algorithm 1.
**Algorithm 1.** Artificial Bee Colony Optimization Algorithm1: **for** the n-th (n<N) RF chain **do**;2: (Step 1) **Initialize:** Generate food sources (current beam-forming vectors $f_{i}^{curr}$) corresponding to *i*th source.;3: (Step 2) **Evaluate** The fitness of beam-forming vectors using (8); Corresponds to achievable rate and select $f_{best}$4: **while**i≠MaxIter5:   (Step 3) **For each Employed Bee**6:   (a) Produce a new beam-forming vector $f_{i}^{new}$ using (9).7:   (b) Evaluate its achievable rate using (8)8:   (c) Choose the one (i.e., $f_{i}^{curr}$ or $f_{i}^{new}$) that achieves higher rate.9:   (Step 4) **For each Onlooker Bee**10:  (a) Select a beam-forming vector $f_{i}^{curr}$ depending on probability value.11:  (b) Produce a new beam-forming vector $f_{i}^{new}$ using (9).12:  (c) Evaluate its achievable rate using (8)13:  (d) Choose the one (i.e., $f_{i}^{curr}$ or $f_{i}^{new}$) that achieves higher rate.]14:  (Step 5) Scout Bees Phase; Randomly Re-Initialize beam-forming vectors whose solutions cannot be improved after pre-determined trials and evaluate their achievable rate.15:  (Step 6) Choose the best beam-forming vector $f_{best}$ based on maximum Achievable Rate.16: **end while**17: (Step 7) Decompose the best beam-forming vector $f_{best}$ into analogue and digital pre-coder
(11)$$a_{n}=e^{j∠(f_{best})}$$
(12)$$d_{n}=\frac{1}{L}{∥f_{best}∥}_{1}$$18: **end for**19: **A** = blkdiag[$a_{1}$, $a_{2}$, … , $a_{N}$]
(13)$$A=\left(\begin{array}{cccc} a_{1} & 0 & \cdots & 0\\ 0 & a_{2} & \cdots & 0\\ \vdots & \vdots & \ddots & \vdots \\ 0 & 0 & \cdots & a_{N} \end{array}\right)$$20: **D** = diag[$d_{1}$, $d_{2}$, … , $d_{N}$]
(14)$$D=\left(\begin{array}{cccc} d_{1} & 0 & \cdots & 0\\ 0 & d_{2} & \cdots & 0\\ \vdots & \vdots & \ddots & \vdots \\ 0 & 0 & \cdots & d_{N} \end{array}\right)$$


Technically, the joint pre-coder ($f_{best}$) that is obtained from the BEE algorithm should be decomposed into $a_{n}$ and $d_{n}$.

(15) $$a_{n}=e^{j∠(f_{best})}$$

where $∠(f_{best})$ represent the phase vector of $f_{best}$ and all the elements of $a_{n}$ share the same amplitude and now $d_{n}$ is obtained as

(16) $$d_{n}=\frac{1}{L}{∥f_{best}∥}_{1}$$

**Figure 2:**
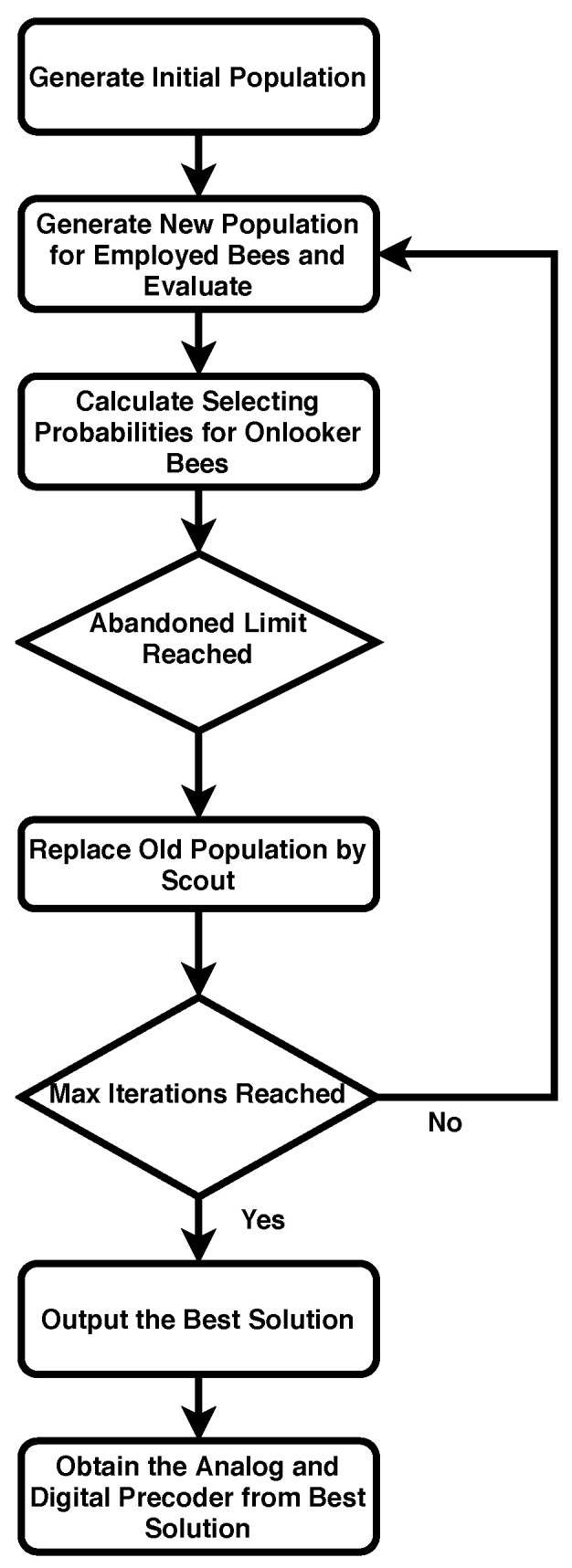
Flow chart of Algorithm.

After obtaining the pre-coder vector for the *n*-th RF chain, the same algorithms are applied to other chains. Finally, a block diagonal A and D matrices are obtained for complete system. The flow chart of Algorithm 1 is shown in Figure 2.

### 3.2. Particle Swarm Optimization

PSO is another effective evolutionary algorithm, which is based on the swarm food searching dynamics. The agents in the swarm iteratively search the solution space to determine the near optimal solution.

We now highlight the way PSO algorithm selects the pre-coding vector $f_{n}$. The agents in the swarm represent the pre-coding vectors. In each iteration of PSO, the direction of each agent representing the pre-coding vector $f_{n}$ is directed towards the best location based on the fitness function (i.e., achievable rate) evaluated using (8).

The algorithm first initializes *E* agents with random positions $f_{n1}\left(0\right)$, $f_{n2}\left(0\right)$, …, $f_{nE}\left(0\right)$. After that, the velocity of all the *E* agents $v_{n1}\left(0\right)$, …, $v_{nE}\left(0\right)$ is randomly initialized. Note that the dimensions of $f_{n}$ and $v_{n}$ are L×1. After initialization, the following iterative process is carried out to update the velocity and position of *E*-th agent. Here c1, c2, $w_{1}$ and $w_{2}$ are tuning parameters and can be chosen to optimize the results.

(17) $$\begin{array}{ccc} v_{n}\left(\alpha +1\right) & = & v_{n}\left(\alpha \right)+c1w_{1}⊙\left(f_{nbest}\left(\alpha \right)-f_{ncurrent}\left(\alpha \right)\right)\\ & + & c2w_{2}⊙\left(f_{best}-f_{ncurrent}\left(\alpha \right)\right) \end{array}$$

(18) $$f_{n}\left(\alpha +1\right)=f_{n}\left(\alpha \right)+v_{n}\left(\alpha +1\right)$$

where $f_{nbest}$ is the best pre-coding vector for *n*-th specific agent and $f_{best}$ is the best pre-coding vector among all *E* agents. The complete algorithm is summarized in Algorithm 2.
**Algorithm 2.** Particle Swarm Optimization Algorithm1: **for** the *n*-th (n<N) RF chain **do**;2: (Step 1) **Initialize:** Generate swarm positions (current beam-forming vectors $f_{n}$) corresponding to *n*th agent;3: (Step 2) **Initialize:** Generate swarm velocities ($v_{n}$) corresponds to *n*th agent;4: (Step 3) Evaluate the fitness (achievable rate) of each agent using (8) and determine the global best $f_{best}$5: **while** i≠MaxIter**do**6:   (Step 4) **For each agent in the swarm**7:   (a) update the velocity and position of each agent as per the following iterative procedure
(19)$$\begin{array}{ccc} v_{n}\left(\alpha +1\right) & = & v_{n}\left(\alpha \right)+c1w_{1}⊙\left(f_{nbest}\left(\alpha \right)-f_{ncurrent}\left(\alpha \right)\right)\\ & + & c2w_{2}⊙\left(f_{best}-f_{ncurrent}\left(\alpha \right)\right) \end{array}$$
(20)$$f_{n}\left(\alpha +1\right)=f_{n}\left(\alpha \right)+v_{n}\left(\alpha +1\right)$$8:   (b) Evaluate the fitness or achievable rate of each agent using (8)9:   (c) if(agent current rate > agent best rate) (agent best fitness ($f_{nbest}$) = agent current fitness)10:  (Step 5) **end For**11:  (Step 6) Choose the best beam-forming vector $f_{best}$ based on maximum Achievable Rate.12: **end while**13: **end for**14: (Step 7) Decompose the best beam-forming vector $f_{best}$ into analogue and digital pre-coder
(21)$$a_{n}=e^{j∠(f_{best})}$$
(22)$$d_{n}=\frac{1}{L}{∥f_{best}∥}_{1}$$15: **A** = blkdiag[$a_{1}$, $a_{2}$, … , $a_{N}$]
(23)$$A=\left(\begin{array}{cccc} a_{1} & 0 & \cdots & 0\\ 0 & a_{2} & \cdots & 0\\ \vdots & \vdots & \ddots & \vdots \\ 0 & 0 & \cdots & a_{N} \end{array}\right)$$16: **D** = diag[$d_{1}$, $d_{2}$, … , $d_{N}$]
(24)$$D=\left(\begin{array}{cccc} d_{1} & 0 & \cdots & 0\\ 0 & d_{2} & \cdots & 0\\ \vdots & \vdots & \ddots & \vdots \\ 0 & 0 & \cdots & d_{N} \end{array}\right)$$


The joint pre-coder is decomposed into analog pre-coder and digital pre-coder using the same procedure as mentioned followed by the BEE algorithm.

Genetic Algorithm (GA) [45] and Cuckoo Search [45] are also popular evolutionary algorithms, however due to space limitations only the BEE algorithm is discussed in detail. In the next section, we will discuss simulations and results in detail.

## 4. Simulations and Results

Simulations are performed for a partially connected hybrid structure, where each RF chain is connected to *L* antennas. The system carrier frequency is assumed to be 28 GHz. The number of receive antennas is set to either 16 and 36, whereas the number of transmit antennas is set to either 64, 144 or 256. The number of RF chains $N_{rf}$ and the number of transmit data streams $N_{s}$ is assumed to be equal and set to make $L=N_{T}/N_{rf}$ an integer value. The channel matrix is generated according to [28]. Analysis for variable number of iterations ($N_{iter}$) of evolutionary algorithms is also performed to evaluate the saturation point of the algorithm. The population ($N_{pop}$) of evolutionary algorithms is set to 70. For a mmW channel, the total number of paths (*K*) is composed of clusters and rays, i.e., $\left(K=N_{cl}\times N_{ray}\right)$, where $N_{cl}$ and $N_{rays}$ are set to 5 and 10, respectively. We will first discuss the analysis for spectral efficiency and the effect of transmit antennas for SIC, BEE and PSO based algorithms. Secondly, we will evaluate the effect of RF chains and the effect of evolutionary algorithm parameters, i.e., $N_{pop}$ and $N_{iter}$ on the spectral efficiency. Finally, we will evaluate the performance of the proposed BEE algorithm in the case of imperfect channel state information.

### 4.1. Spectral Efficiency Analysis for Variable Transmit Antennas

**Figure 3:**
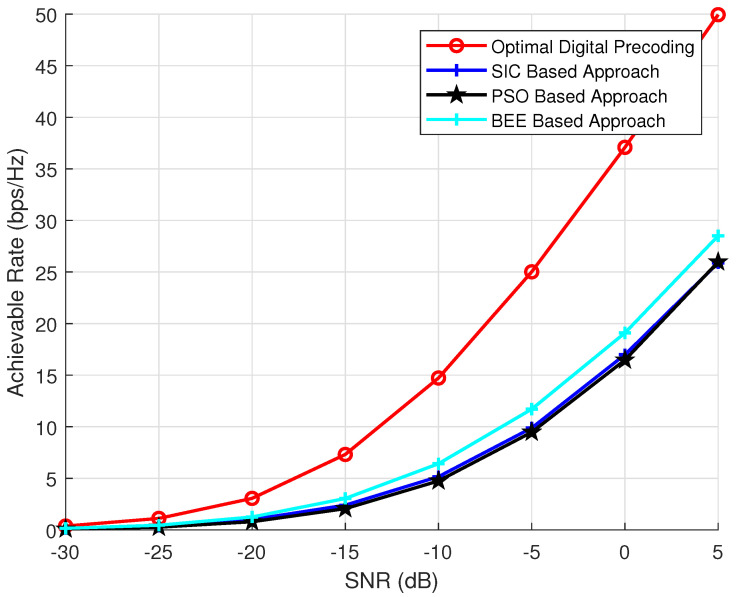
Simulations using $N_{T}$ = 64, $N_{R}$ = 16, $N_{rf}$ = 8.

**Figure 4:**
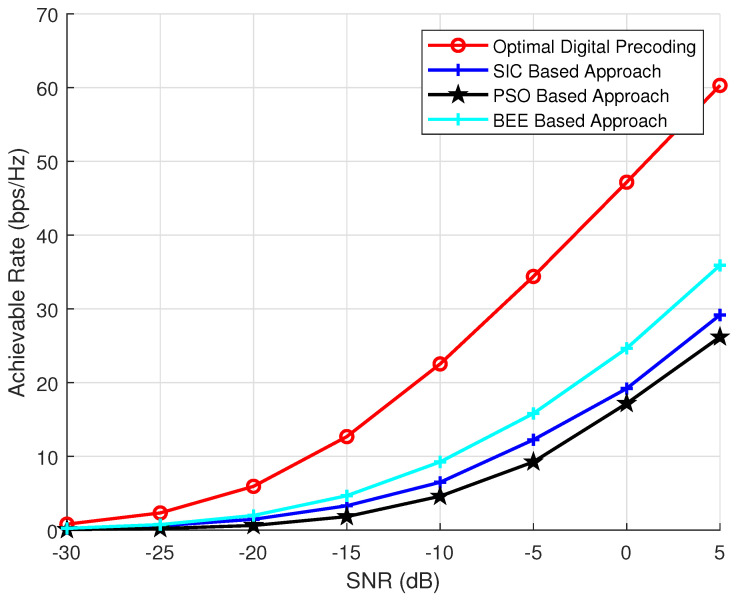
Simulations using $N_{T}$ = 144, $N_{R}$ = 36, $N_{rf}$ = 8.

**Figure 5:**
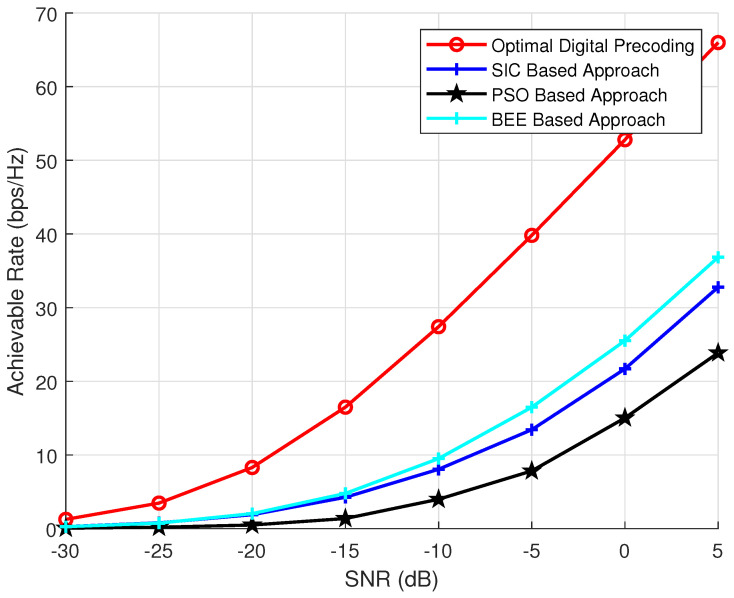
Simulations using $N_{T}$ = 256, $N_{R}$ = 36, $N_{rf}$ = 8.

To investigate the effect of transmit antennas on the performance of BEE, SIC and PSO based algorithms, we have performed analysis by varying the number of transmit antennas as 64, 144 and 256. The parameters $N_{R}$ and $N_{rf}$ are kept constant and set to 36 and 8, respectively. The number of iterations and the population of evolutionary algorithms is also kept as 70. The simulation results (Figure 3, Figure 4 and Figure 5) verify the superiority of BEE based approach over SIC and PSO based schemes. Our proposed scheme is performing satisfactory for both MIMO and massive MIMO architectures. The BEE algorithm performing the best among other evolutionary algorithms is due to the fact that those agents which cannot be improved after a predetermined number of trials are abandoned and converted into a new random agent (i.e., new random beam-forming vector), which may approach the optimal performance. The agents contributing to low spectral efficiency are more often discarded and converted to new agents. Such abandonment of agents is not performed by any other evolutionary algorithm, which makes BEE the most efficient.

### 4.2. Spectral Efficiency Analysis for Variable RF Chains and Evolutionary Algorithm Parameters

**Figure 6:**
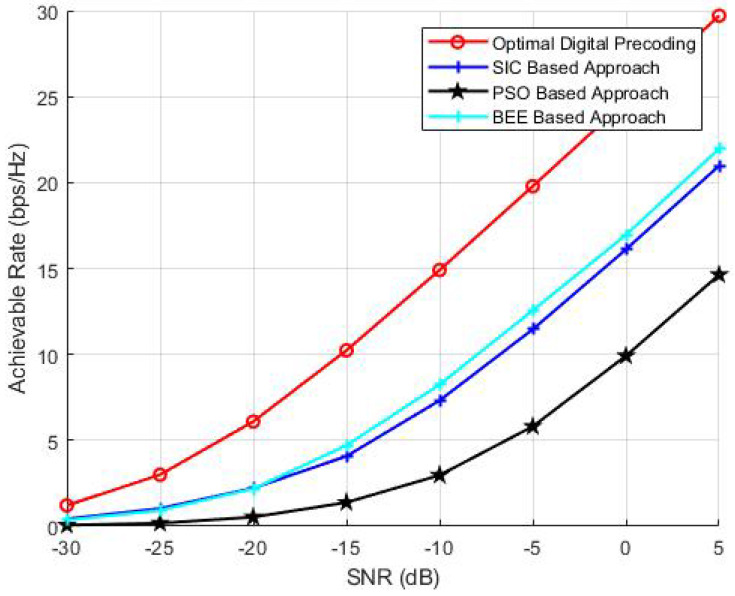
Simulations using $N_{T}$ = 144, $N_{R}$ = 36, $N_{rf}$ = 3.

**Figure 7:**
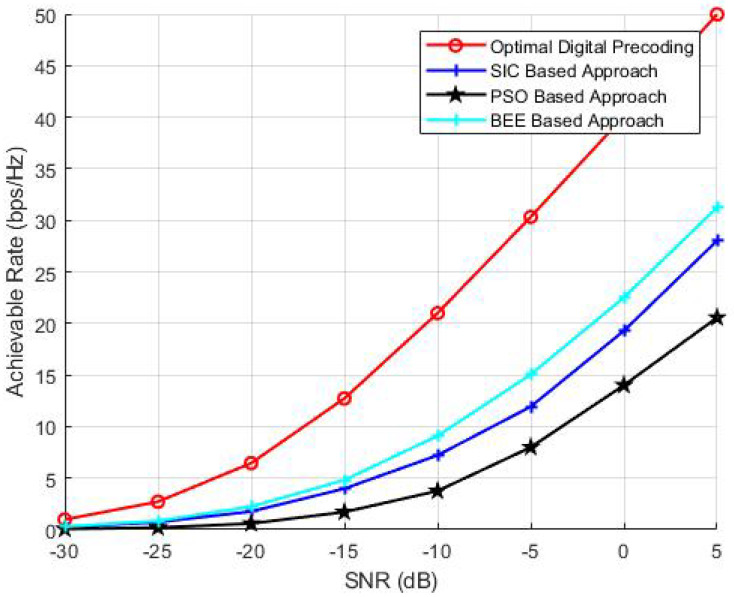
Simulations using $N_{T}$ = 144, $N_{R}$ = 36, $N_{rf}$ = 6.

**Figure 8:**
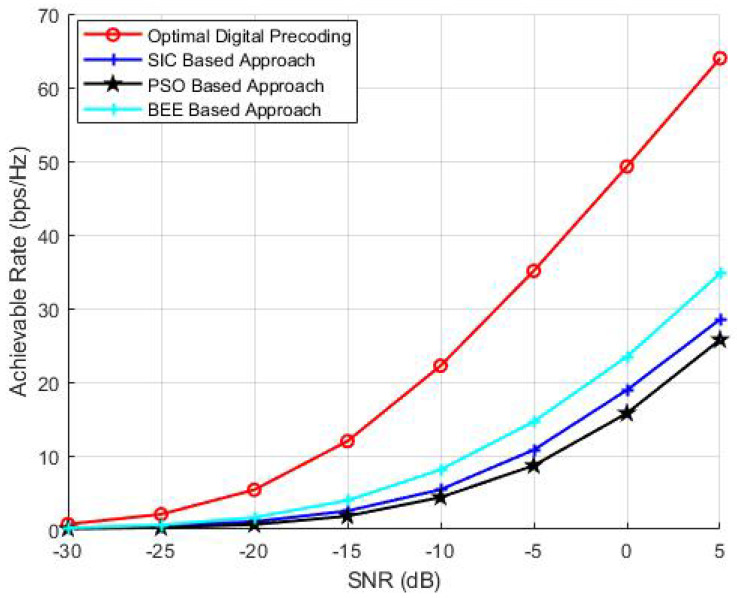
Simulations using $N_{T}$ = 144, $N_{R}$ = 36, $N_{rf}$ = 9.

**Figure 9:**
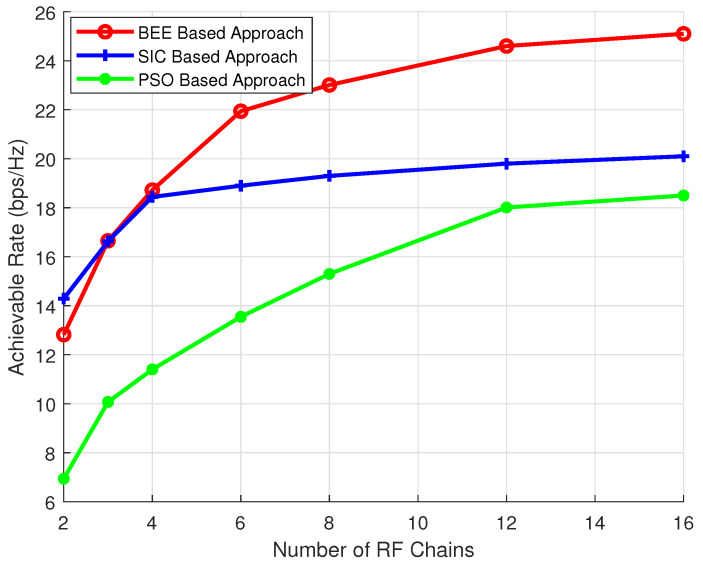
Simulation for varying number of RF chains.

The effect of variable number of RF chains on the performance of algorithms is discussed in this section. $N_{T}$ is kept as 144, $N_{R}$ is kept as 36, the simulations are performed for variable number of RF chains ($N_{rf}$ = 3, 6 and 9). The results (Figure 6, Figure 7 and Figure 8) show that the proposed scheme outperforms SIC based and PSO based pre-coding irrespective of the number of RF chains. Moreover, the performance gap between the BEE algorithm and the SIC based algorithm increases with the number of RF chains. Figure 9 validates the performance superiority of the proposed algorithm when the RF chains increased.

Further, we investigate the spectral efficiency performance of the BEE based algorithm with variable number of iterations and population. Since the efficiency of evolutionary algorithms is dependent on the number of populations and iterations, we have to determine the suitable values for these parameters and determine the saturation point of the algorithm.

**Figure 10:**
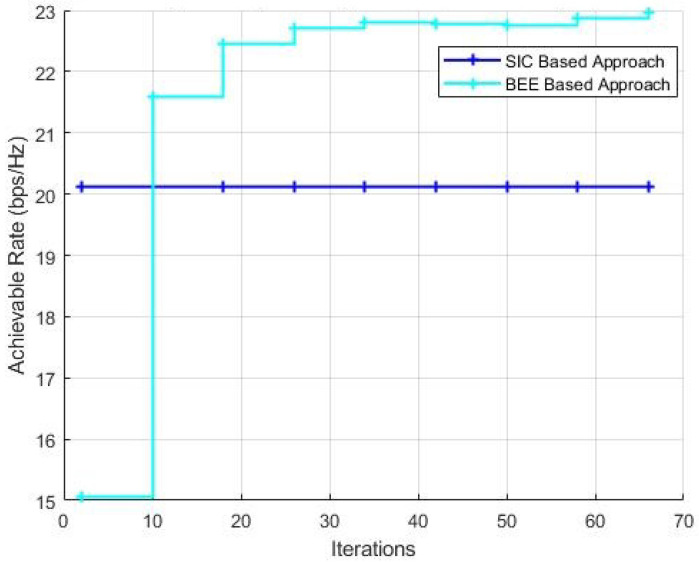
Simulations with $N_{T}$ = 144, $N_{R}$ = 36, $N_{rf}$ = 9, population = 70.

**Figure 11:**
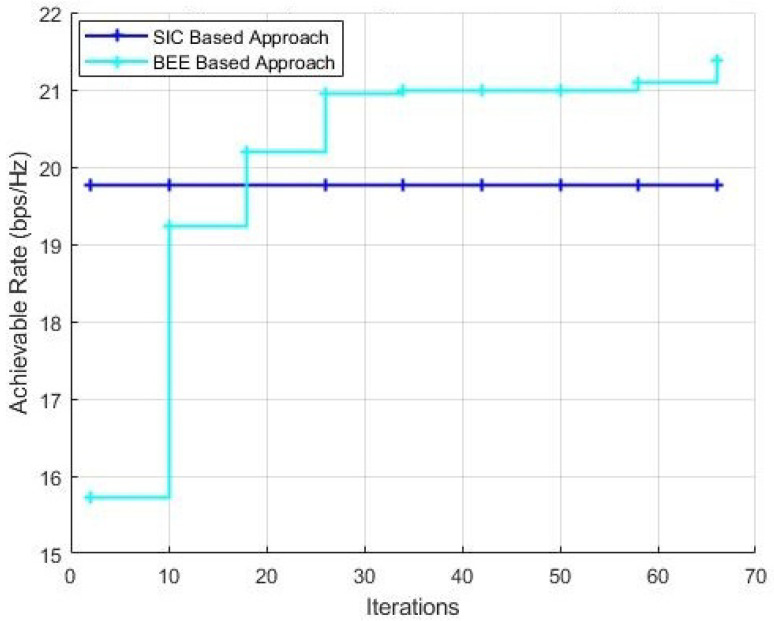
Simulations with $N_{T}$ = 144, $N_{R}$ = 36, $N_{rf}$ = 9, population = 20.

Figure 10 and Figure 11 show the performance comparison with a variable number of iterations for BEE algorithm with the population kept as 70 and 20, respectively. It can be verified that when the population is kept as 70, the BEE based algorithm outclasses the SIC based algorithm within just 10 iterations of the algorithm. When the population is decreased to 20, the optimal number of iterations to outperform the SIC based algorithm is 20.

### 4.3. Performance Analysis with Imperfect CSI

**Figure 12:**
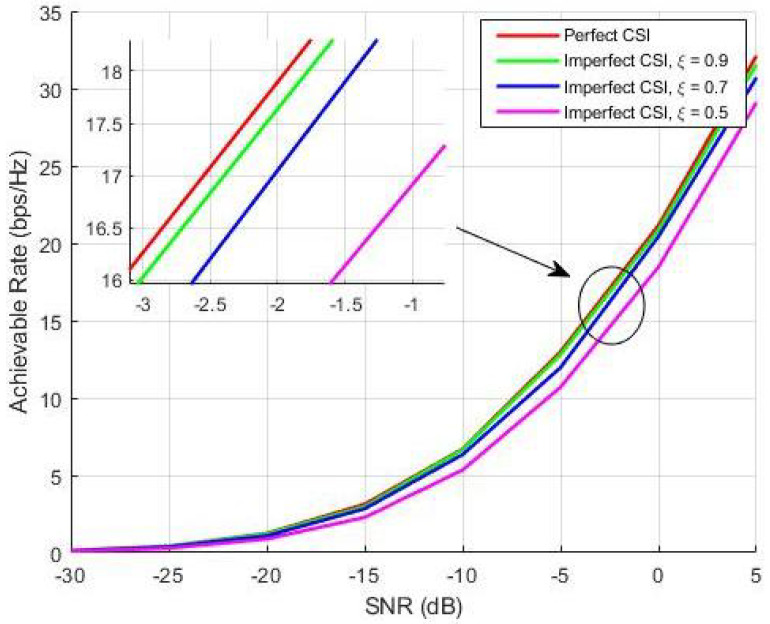
Perfect channel state information (CSI) vs Imperfect CSI.

In this section, we performed the analysis of uncertainties effecting the system channel. Let $\hat{H}$ represent the estimated channel matrix with the imperfect CSI and be modeled as [26]

(25) $$\hat{H}=\xi H+\sqrt{1-\xi^{2}}E$$

where the channel matrix with the perfect CSI is denoted by H, the CSI precision value is denoted by ξ∈ [0,1] and E represents the error matrix having distribution i.i.d CN (0,1). Figure 12 analyzes the imperfect CSI for a mmW massive MIMO architecture with $N_{T}=144,N_{R}=36,N_{RF}=9$. Results verify that the system adopting BEE pre-coding is not overly sensitive to CSI precision. The results also show that the achievable rate of proposed BEE based pre-coding system with the CSI precision value ξ=0.9 is very close to that of the case of perfect CSI. Furthermore, even when the CSI precision value is degraded (i.e., ξ=0.5), the proposed BEE based hybrid pre-coding system is still performing well.

### 4.4. Complexity Analysis

Evolutionary algorithms can generally attain lower computational complexity than the conventional algorithms [33]. As the evolutionary algorithms are iterative, their computational complexity depends on the loop iterations and fitness function. Evolutionary algorithms are most efficient (both in terms of spectral efficiency and computational complexity) when RF chains are high. This is because the evolutionary algorithms are applied on each RF chain separately and when the number of antennas per RF chain is less, the complexity of fitness function is reduced. Hence, making the evolutionary algorithms more efficient. As the number of RF chains increases the number of Antennas per RF chains (L) decreases. Since the computational complexity of evolutionary algorithm is largely dependent on the evaluation of fitness function which is a function of L, the decrease in L makes the computation of fitness function less extensive. On the other hand, the computational complexity of the SIC based approach has a quadratic dependence on the number of RF chains, hence an increases in the number of RF chains significantly increases the computational complexity of the SIC algorithm. Furthermore, the increase in the number of RF chains decreases the computational complexity of evolutionary algorithms since they only take into account the current RF chain while determining the pre-coder and do not involve the interface cancellation of other RF chains as in the case of SIC based approach. This greatly reduces the computational complexity since only *L* antennas are taken into account at a time instead of all $N_{t}$ antennas. Note that the error propagation is an issue with SIC based approach. The error introduced while computing any particular pre-coder will propagate and effect the computation of subsequent pre-coders and thus making SIC based approach inefficient. The computational complexity in terms of flops of evolutionary algorithms is $O(N_{pop}N_{iter}N_{rf}L^{2}M^{2})$ and for SIC based approach comes out to be $O(2MN_{s}\left(N_{t}^{2}\left(1+N_{s}M\right)\right))$ [26].

We have considered computational complexity as a measure of the total number of flops required by algorithm instead of time complexities since the later is dependent on machine type and coding style. Number of flops is a more specific representation of any algorithm’s computational complexity.

## 5. Conclusions

In this paper, evolutionary algorithm based method for a hybrid pre-coding system was proposed for partially connected antenna array architecture for a mmW Massive MIMO system. Simulation results showed that BEE algorithm based solution was able to achieve a higher achievable rate than other algorithms owing to the inherent capability of the algorithm to discard inefficient solutions and replace them with new ones. The characterization of the proposed scheme on the basis of the number of RF chains was performed. The results verify that the evolutionary algorithm based solutions are more efficient than conventional techniques irrespective of the number of RF chains or transmit antennas. The BEE algorithm always outperforms other algorithms in terms of achievable rate with a limited number of tuning parameters. 

## References

[1] Rappaport T., Sun S., Mayzus R., Zhao H., Azar Y., Wang K., Wong G., Schulz J., Samimi M., Gutierrez F. (2013). Millimeter wave mobile communications for 5G cellular: It will work!. IEEE Access.

[2] Rangan S., Rappaport T., Erkip E. (2014). Millimeter-wave cellular wireless networks: Potentials and challenges. Proc. IEEE.

[3] Sun S., Rappaport T.S., Heath R.W., Nix A., Rangan S. (2014). MIMO for millimeter-wave wireless communications: Beamforming, spatial multiplexing, or both?. IEEE Commun. Mag..

[4] Hoydis J., Brink S.T., Debbah M. (2013). Massive MIMO in the UL/DL of cellular networks: How many antennas do we need?. IEEE J. Sel. Areas Commun..

[5] Larsson E.G., Edfors O., Tufvesson F., Marzetta T.L. (2014). Massive MIMO for next generation wireless systems. IEEE Commun. Mag..

[6] Andrews J.G., Buzzi S., Choi W., Hanly S.V., Lozano A., Soong A.C.K., Zhang J.C. (2014). What will 5G be?. IEEE J. Sel. Areas Commun..

[7] Boccardi F., Heath R.W., Lozano A., Marzetta T.L., Popovski P. (2014). Five disruptive technology directions for 5G. IEEE Commun. Mag..

[8] Björnson E., Sanguinetti L., Kountouris M. (2015). Deploying dense networks for maximal energy efficiency: Small cells meet massive MIMO. IEEE J. Sel. Areas Commun..

[9] Li C., Zhang J., Letaief K.B. (2014). Throughput and energy efficiency analysis of small cell networks with multi-antenna base stations. IEEE Trans. Wirel. Commun..

[10] Roh W., Seol J.Y., Park J., Lee B., Lee J., Kim Y., Cho J., Cheun K., Aryanfar F. (2014). Millimeter-wave beamforming as an enabling technology for 5G cellular communications: Theoretical feasibility and prototype results. IEEE Commun. Mag..

[11] Bai T., Alkhateeb A., Heath R. (2014). Coverage and capacity of millimeter-wave cellular networks. IEEE Commun. Mag..

[12] Pi Z., Khan F. (2011). An introduction to millimeter-wave mobile broadband Systems. IEEE Commun. Mag..

[13] Marzetta T.L. (2010). Noncooperative cellular wireless with unlimited numbers of base station antennas. IEEE Trans. Wirel. Commun..

[14] Ngo H., Larsson E., Marzetta T. (2012). Energy and spectral efficiency of very large multiuser MIMO systems. IEEE Trans. Commun..

[15] Rusek F., Persson D., Lau B.K., Larsson E.G., Marzetta T.L., Edfors O., Tufvesson F. (2013). Scaling up MIMO: Opportunities and challenges with very large arrays. IEEE Signal Process. Mag..

[16] Wei L., Hu R.Q., Qian Y., Wu G. (2014). Key elements to enable millimeter wave communications for 5G wireless systems. IEEE Wirel. Commun..

[17] Alkhateeb A., Mo J., González-Prelcic N., Heath R. (2014). MIMO precoding and combining solutions for millimeter-wave systems. IEEE Commun. Mag..

[18] Yin B., Abu-Surra S., Xu G., Henige T., Pisek E., Pi Z., Cavallaro J.R. High-throughput beamforming receiver for millimeter wave mobile communication. Proceedings of the 2013 IEEE Global Communications Conference (GLOBECOM’13).

[19] Abbas W.B., Gomez-Cuba F., Zorzi M. (2017). Millimeter Wave Receiver Efficiency: A Comprehensive Comparison of Beamforming Schemes With Low Resolution ADCs. IEEE Trans. Wirel. Commun..

[20] Han S., Chih-Lin I., Xu Z., Rowell C. (2015). Large-scale antenna systems with hybrid precoding analog and digital beamforming for millimeter wave 5G. IEEE Commun. Mag..

[21] El Ayach O., Rajagopal S., Abu-Surra S., Pi Z., Heath R.W. (2014). Spatially sparse precoding in millimeter wave MIMO systems. IEEE Trans. Wirel. Commun..

[22] Chen C. (2015). An iterative hybrid transceiver design algorithm for millimeter wave MIMO systems. IEEE Wirel. Commun. Lett..

[23] Lee Y.Y., Wang C.H., Huang Y.H. (2015). A hybrid RF/baseband precoding processor based on parallel-index-selection matrix-inversion-bypass simultaneous orthogonal matching pursuit for millimeter wave MIMO systems. IEEE Trans. Signal Process..

[24] Kim C., Son J.S., Kim T., Seol J.Y. On the hybrid beamforming with shared array antenna for mmWave MIMO-OFDM systems. Proceedings of the 2014 IEEE Wireless Communications and Networking Conference (WCNC).

[25] Khalid F. (2019). Hybrid Beamforming for Millimeter Wave Massive Multiuser MIMO Systems Using Regularized Channel Diagonalization. IEEE Wirel. Commun. Lett..

[26] Gao X., Dai L., Han S., Chih-Lin I., Heath R.W. (2016). Energy-efficient hybrid analog and digital precoding for mmWave MIMO systems with large antenna arrays. IEEE J. Sel. Areas Commun..

[27] Dai L., Gao X., Quan J., Han S., Chih-Lin I. Near-optimal hybrid analog and digital precoding for downlink mmwave massive mimo systems. Proceedings of the 2015 IEEE International Conference on Communications (ICC).

[28] Zhang D., Wang Y., Li X., Xiang W. (2018). Hybridly Connected Structure for Hybrid Beamforming in mmWave Massive MIMO Systems. IEEE Trans. Commun..

[29] Zhang J., Huang Y., Yu T., Wang J., Xiao M. (2018). Hybrid Precoding for Multi-Subarray Millimeter-Wave Communication Systems. IEEE Wirel. Commun. Lett..

[30] Alluhaibi O., Ahmed Q.Z., Wang J., Zhu H. Hybrid digital-to-analog precoding design for mm-wave systems. Proceedings of the 2017 IEEE International Conference on Communications (ICC).

[31] Yu X., Shen J.C., Zhang J., Letaief K.B. (2016). Alternating Minimization Algorithms for Hybrid Precoding in Millimeter Wave MIMO Systems. IEEE J. Sel. Top. Signal Process..

[32] Alluhaibi O., Nair M., Hazzaa A., Mihbarey A., Wang J. 3D Beamforming for 5G Millimeter Wave Systems Using Singular Value Decomposition and Particle Swarm Optimization Approaches. Proceedings of the International Conference on Information and Communication Technology Convergence (ICTC).

[33] Alluhaibi O., Ahmed Q.Z., Pan C., Zhu H. Hybrid Digital-to-Analog Beamforming Approaches to Maximise the Capacity of mm-Wave Systems. Proceedings of the IEEE 85th Vehicular Technology Conference (VTC Spring).

[34] Xie Y., Li B., Yan Z., Fan J., Yang M. (2019). A General Hybrid Precoding Method for mmWave Massive MIMO Systems. Radio Eng..

[35] Liu X., Li X., Cao S., Deng Q., Ran R., Nguyen K., Tingrui P. (2019). Hybrid Precoding for Massive mmWave MIMO Systems. IEEE Access.

[36] Kolawole O.Y., Biswas S., Singh K., Ratnarajah T. (2020). Transceiver Design for Energy-Efficiency Maximization in mmWave MIMO IoT Networks. IEEE Trans. Green Commun. Netw..

[37] Zhao P., Wang Z. (2018). Hybrid Precoding for Millimeter Wave Communications With Fully Connected Subarrays. IEEE Commun. Lett..

[38] Majidzadeh M., Kaleva J., Tervo N., Pennanen H., Tölli A., Latva-Aho M. Rate Maximization for Partially Connected Hybrid Beamforming in Single-User MIMO Systems. Proceedings of the IEEE 19th International Workshop on Signal Processing Advances in Wireless Communications (SPAWC).

[39] Nair M., Ahmed Q.Z., Wang J., Zhu H. Low-Complexity Hybrid Digital-to-Analog Beamforming for Millimeter-Wave Systems with High User Density. Proceedings of the IEEE 85th Vehicular Technology Conference (VTC Spring).

[40] Alluhaibi O., Ahmed Q.Z. Multi-User Hybrid Precoding and Decoding Design for mm-Wave Large Antenna Systems. Proceedings of the IEEE 87th Vehicular Technology Conference (VTC Spring).

[41] Elbir A.M., Mishra K.V. (2020). Joint Antenna Selection and Hybrid Beamformer Design Using Unquantized and Quantized Deep Learning Networks. IEEE Trans. Wirel. Commun..

[42] Choi J., Lee N., Hong S., Caire G. (2020). Joint User Selection, Power Allocation, and Precoding Design With Imperfect CSIT for Multi-Cell MU-MIMO Downlink Systems. IEEE Trans. Wirel. Commun..

[43] Han S., Chih-Lin I., Xu Z., Wang S. (2014). Reference signals design for hybrid analog and digital beamforming. IEEE Commun. Lett..

[44] Alkhateeb A., El Ayach O., Leus G., Heath R.W. (2014). Channel estimation and hybrid precoding for millimeter wave cellular systems. IEEE J. Sel. Top. Signal Process..

[45] Yang X.-S. (2014). Nature-Inspired Optimization Algorithms.

